# Comparative Cytogenetics Analysis of *Chlamys farreri*, *Patinopecten yessoensis,* and *Argopecten irradians* with *C_0_t*-1 DNA by Fluorescence *In Situ* Hybridization

**DOI:** 10.1155/2011/785831

**Published:** 2011-07-07

**Authors:** Li-Ping Hu, Wen-Cong Shang, Yan Sun, Shan Wang, Xiao-Liang Ren, Xiao-Ting Huang, Zhen-Min Bao

**Affiliations:** Key Laboratory of Marine Genetics and Breeding (MGB), College of Marine Life Sciences, Ocean University of China, Ministry of Education, Qingdao 266003, China

## Abstract

The chromosomes of *Chlamys farreri*, *Patinopecten yessoensis,* and *Argopecten irradians* were studied by FISH using *C. farreri C_0_t*-1 DNA probes. The results showed that *C_0_t*-1 DNA signals spread on all chromosomes in the three scallops, whereas signal density and intensity were different strikingly. Clustering brighter signals presented in the centromeric and telomeric regions of most *C. farreri* chromosomes, and in the centromeric or pericentromeric regions of several *P. yessoensis* chromosomes. Comparative analysis of the mapping indicated a relatively higher homology in the repetitive DNA sequences of the genome between *C. farreri* and *P. yessoensis* than that between *C. farreri* and *A. irradians*. In addition, FISH showed that the distribution of *C_0_t*-1 DNA clustering signals in *C. farreri* displayed completely similar signal bands between homologous chromosomes. Based on the *C_0_t*-1 DNA fluorescent bands, a more exact *karyotype* of *C. farreri* has been obtained. In this study, the comparative analysis based on *C_0_t*-1 DNA provides a new insight into the chromosomal reconstructions during the evolution process, and it is helpful for understanding an important source of genomic diversity, species relationships, and genome evolution.

## 1. Introduction

The scallop family, Pectinidae, including more than 300 living species recognized in worldwide oceans, is one important fauna of bivalve not only at commercial and ecological levels, but also in terms of biological evolution and basic biology research. Given their importance, scallops have been the subject of much research. In the last few decades, a wide range of systematic studies have been conducted in accordance with morphological features [1–3] and molecular phylogeny [4, 5]. In addition, cytogenetic methods also have played important roles in the analysis of karyotype evolution. In recent years, cytogenetic analysis by Fluorescence *in situ* hybridization (FISH) has been effectively carried out in Pectinidae, such as localizing repetitive sequences [6–12], and karyotypic evolution by comparison of rRNA and histone H3 gene loci [13, 14]. The cytogenetic data available has indicated great deviation in chromosome number and morphologies among species, suggesting that significant changes in chromosome number and structure have occurred during the evolution of Pectinidae [13]. However, the current cytogenetic evidences on the scallops evolution most focus on comparison of chromosome numbers by karyotype or the locus of a single gene (rRNA or H3 gene) by FISH. The comparative cytogenetics investigations in the genomewide level have not been reported among Pectinidae species.

Comparative cytogenetics, as a powerful tool to study karyotypic variation, is based on accurate chromosome identification. Chromosome banding and FISH techniques have facilitated cytogenetic research for many animal and plant species, unfortunately, chromosome identification remains a challenge in scallop and other bivalve species. The practice of conventional chromosome banding in scallops and oysters confirms its low reliability to identify individual chromosomes [10, 15, 16]. The current researches on the identification of the chromosomes of scallops are on the basis of localization of repetitive sequences by FISH, such as rRNA genes [6, 9, 13] and histone H3 genes [14]. Furthermore, fosmid clones have been applied to identification of *C. farreri* chromosomes and identified 8 from 19 chromosome pairs [17]. So far, there has not been a successful example to identify all the chromosome pairs of any scallop species.

The genomes of eukaryotic species contain numerous types of highly or moderately repetitive DNA elements. It has been showen that the variation in genome size is largely caused by differences in the amount of repetitive DNA sequences. The repetitive DNA sequences used as the molecular marker play significant roles in comparative genomics study, especially the research on structure and function of species genomes and the evolution of chromosomes. For example, the satellite repeat sequences were exploited for genetic linkage maps construction [18] and variety identification [19, 20]. The *in situ* investigation of repetitive DNA sequences adds new informative characters useful in comparative genomics at chromosomal level and provides insights into the evolutionary relationships among scallops [13, 14], as well as the hybridization of satellite DNAs has contributed to the relationship among fish species and their karyotypic diversification [21–23].

*C_0_t*-1 DNA is enriched with highly and moderately repetitive DNA sequences, which have been widely used as a blocking agent to inhibit hybridization of repeats present within DNA probes. In some plant species, *C_0_t*-1 DNA has also been used for karyotyping and comparative analysis of genomes by FISH [24, 25]. Zhikong scallop (*Chlamys farreri* Jones et Preston, 1904), Yesso scallop (*Patinopecten yessoensis* Jay, 1857), and Bay scallop (*Argopecten irradians* Lamarck, 1819) are important commercial species in China. *C. farreri *distribute naturally in the northern seacoasts of China. *P. yessoensis* and* A. irradians* were introduced to China from their original distribution areas—Hokkaido, Japan in 1980 [26] and North America in 1982 [27], respectively. To analyze the genome structure and detect chromosome evolution of these three species, we used *C_0_t*-1 DNA of *C. farreri* (called CF* C_0_t*-1 DNA for short) as probes for *in situ *hybridization on mitotic metaphase chromosomes of these scallops and analyzed the signals distribution of the repetitive sequences in the three genomes. In addition, since these DNA sequences dispersed in the whole genome, we tried to identify individual pairs from all the chromosome pairs of *C. farreri* with the hybridization signal bands.

## 2. Materials and Methods

### 2.1. Scallop Materials and Chromosome Preparations

Sexually mature scallops (*C. farreri, A. irradians, *and* P. yessoensis*) were obtained from a hatchery in Shandong Province, China. The chromosome preparations were performed following the method of Zhang et al. [14]. Briefly, the larvae were treated with colchicine (0.01%) for 2 h at room temperature and KCl (0.075 M) for 30 min, then fixed in Carnoy's fixative (methanol : glacial acetic acid = 3 : 1 v/v) for three times (15 min each). The fixed larvae were dissociated in 50% acetic acid, and then the cell suspension was dropped onto hot-wet slides and air-dried. 

### 2.2. Genomic DNA Extraction and Preparation of CF *C_0_t*-1 DNA

The total genomic DNA extraction was carried out by the standard phenol-chloroform procedure using adductor muscle [28]. CF *C_0_t*-1 DNA was prepared according to the procedure described by Zwick et al. [29] with slightly modified. In brief, genomic DNA was diluted to a concentration of 600 ng/*μ*l in 0.3 M NaCl and was sonicated to 100*–*1500 bp DNA fragments. The shearing DNA was denatured in 95°C bath water for 10 min, and then annealed in 65°C bath water for the required time which was calculated according to the formula *C_0_t*-1 = mol/L × Ts. After the tube was put into ice water for 2 min, the appropriate amount of S1 nuclease (1 U/*μ*g DNA) was added, and reaction at 23°C for 30 min. Finally, the reaction was stopped by adding appropriate amount of EDTA (final concentration of 25 mM). Then the DNA was extracted by Tris-equilibrated phenol, deposited by 2.5 volumes of absolute ice-ethanol and washed with prechilled 70% ethano1, then air-dried and resuspended in TE buffer. CF *C_0_t*-1 DNA was stored in *−*20°C after quantitative analysis.

### 2.3. Probe Labeling and FISH

CF* C_0_t*-1 DNA was labeled with digoxigenin-11-dUTP by nick translation following the manufacturer's instruction (Roche). The length of the CF* C_0_t*-1 DNA used as the probe for FISH was between 100 bp and 600 bp, which was estimated by 2% gel electrophoresis.

FISH was carried out according to Huang et al. [8]. Firstly, chromosomes spreads were pretreated with 0.005% pepsin in 10 mM HCl for 10 min, and followed by washing in 2 × SSC twice 5 min at room temperature. Chromosome spreads were then denatured in a mixture containing 75% formamide and 2 × SSC at 76°C for 2-3 min, dehydrated with a chilled ethanol series, 70%, 90%, 100%, 5 min each, and air-dried. The hybridization mix (10 ng/ul probes, 10% dextran sulfate, and 50% deionized formamide in 2 × SSC) was denatured at 95°C for 6-7 min and chilled immediately by putting on ice for at least 10 min. Denatured probe was applied onto the slide and DNA-DNA *in situ* hybridization was carried out in a humidity chamber at 37°C for 12–16 h. Following hybridization, the slides were washed in 2 × SSC at 42°C 5 min, 50% formamide in 2 × SSC at 42°C 10 min, 1 × SSC at 42°C three times (5 min each), and 2 × SSC once for 5 min at room temperature. Fluorescent signals were detected with antidigoxigenin rhodamine (Roche). Chromosomes were counterstained with DAPI (Vector). Slides were observed using a Nikon E-600 epifluorescence microscope equipped with a CCD camera (COHU). The signals were collected using appropriate filter sets and LUCIA software. The karyotype was reconstructed according to the karyotype standard of Levan et al. [30], as well as CF *C_0_t*-1 DNA signal bands patterns using Lucia-FISH Image System.

## 3. Results

**Figure 1:**
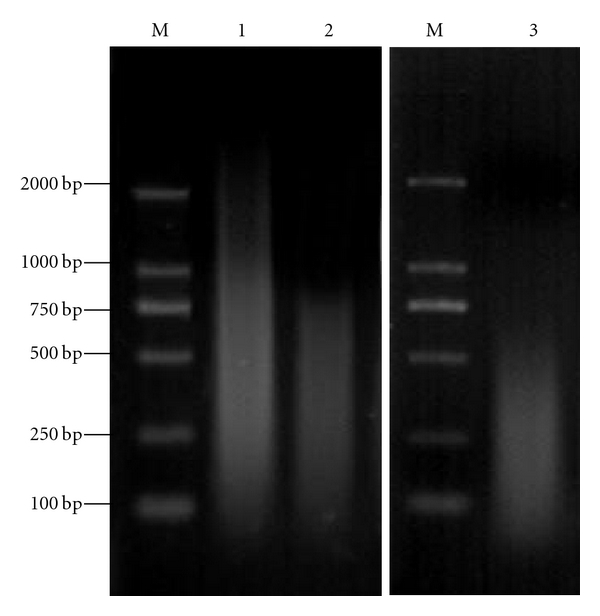
Preparation of *C_0_t*-1 DNA probes of *C. farreri* M DL2000 marker; 1 Shearing genomic DNA; 2 *C_0_t*-1 DNA; 3 Labeled *C_0_t*-1 DNA probes.

The size of the genomic DNA obtained from *C. farreri* was more than 20 kb (data not shown). After being sonicated, the majority of DNA fragments were within the desired size range of 100–1500 bp. Then by reannealing and S1 nuclease digestion, the final size range of CF* C_0_t*-1 DNA is 100–800 bp. The size of CF* C_0_t*-1 DNA probes labeled with digoxigenin-11-dUTP by nick translation was ranged from 100 bp to 600 bp (Figure 1).

**Figure 2:**
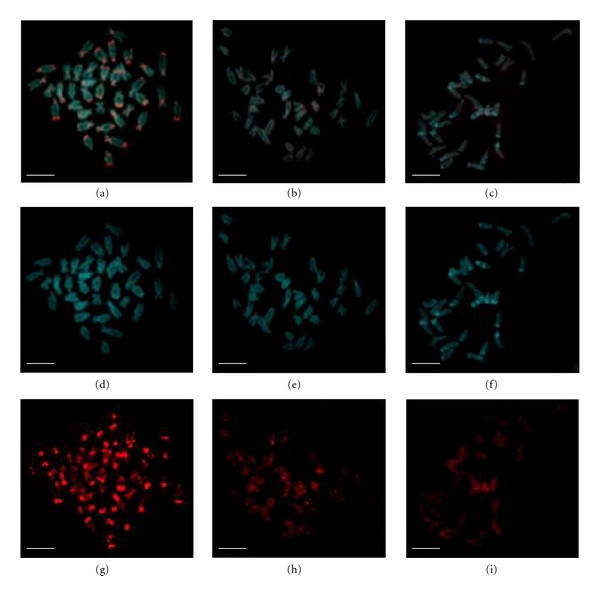
FISH results of *C_0_t*-1 DNA probes to the metaphase chromosomes of *C. farreri*, *P. yessoensis *and *A. irradians. *Bar = 5 *μ*m. (a) and (g), FISH combined image and red hybridization signals image of *C. farreri* probed with its own *C_0_t*-1 DNA; (b) and (h), FISH combined image and red hybridization signals image of *P. yessoensis* probed with *C_0_t*-1 DNA of *C. farreri*; (c) and (i), FISH combined image and red hybridization signals image of *A. irradians* probed with *C_0_t*-1 DNA of* C. farreri*; (d), (e), and (f), DAPI staining images of *C. farreri, P. yessoensis, *and *A. irradians*, respectively.

**Figure 3:**
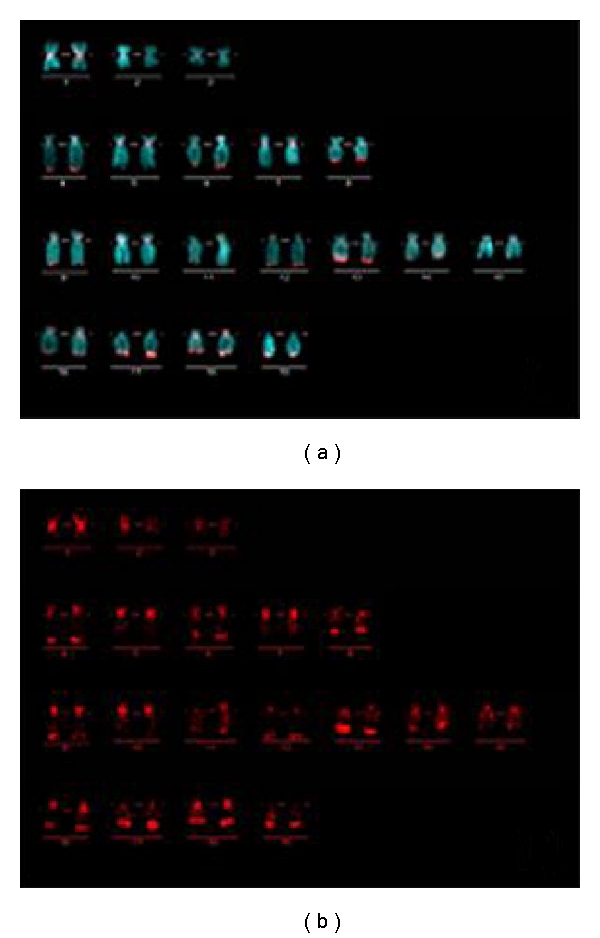
Karyotype of *C. farreri* and corresponding *C_0_t*-1 DNA signal bands.

The labeled CF* C_0_t*-1 DNA was hybridized to the chromosome spreads of *C. farreri, P. yessoensis, *and *A. irradians*. The results were shown in Figure 2. On the metaphase chromosomes of *C. farreri*, the signals of CF* C_0_t*-1 DNA presented in all chromosomes, and the clustering brighter signals of CF* C_0_t*-1 DNA distributed mainly in areas of centromeres, subcentromeres, and near telomeres, and fewer in the middle regions of chromosome arms (Figures 2(a) and 2(g)). In detail, on three pairs of metacentric chromosomes, two pairs of submetacentric chromosome and one pair of subtelocentric chromosomes, the brighter CF* C_0_t*-1 DNA signals mainly concentrated in areas of centromere; on the other three pairs of submetacentric chromosomes and nine pairs of subtelocentric chromosomes, more intensive CF* C_0_t*-1 DNA distributed in areas of centromeres, subcentromeres, and telomeric regions of the long arms; and on another pair of subtelocentric chromosomes, more intensive CF* C_0_t*-1 DNA distributed in telomeric regions of the long arms. From the characteristics of the signals distribution, the repetitive sequences had specific, strong and steady signal bands on chromosomes, and the homologous chromosomes exhibited similar signal bands, so that karyotype analysis could be conducted based on CF* C_0_t*-1 DNA specific fluorescence bands in *C. farreri*. And the karyotype result was shown in Figure 3. Based on the karyotype picture, the signals were much brighter in centromeres areas of the chromosome pairs 1, 5, 7, 9, 10, 16 and 18, and telomeric regions on the long arms of the chromosome pairs 4, 8, 13, 16, 17, 18 and 19 (Figure 3). On the metaphase chromosomes of *P. yessoensis*, the signals of CF* C_0_t*-1 DNA were also detected in all chromosomes, whereas, the brighter clustering signals of CF* C_0_t*-1 DNA could be seen on several chromosomes at centromeric and pericentromeric regions, or near centromeric regions of the long arms (Figures 2(b) and 2(h)). Furthermore, on one or two pairs of subtelocentric chromosomes, the brighter CF* C_0_t*-1 DNA signals mainly concentrated in areas of centromeric and pericentromeric regions; on another pair of subtelocentric chromosomes, more intensive CF* C_0_t*-1 DNA distributed near centromeric regions of the long arms; on the remaining chromosomes, the signals of CF* C_0_t*-1 DNA were dispersed. In contrast, no obviously clustering brighter signals of CF* C_0_t*-1 DNA regions were shown and the signals were dispersed on chromosomes of *A. irradians* (Figures 2(c) and 2(i)). 

Overall, according to the FISH images, the signals of CF* C_0_t*-1 DNA were detected in all chromosomes of the three species, whereas signal density and intensity were different strikingly. The signals of CF* C_0_t*-1 DNA in *C. farreri* appeared the most intensive and brightest, followed by which in *P. yessoensis*, and the signals of CF* C_0_t*-1 DNA in *A. irradians* were the most sparse and weakest. Additionally, CF* C_0_t*-1 DNA showed the clustering brighter signals region on all chromosomes of *C. farreri*, although they displayed different coverage, brightness, and location on different chromosomes, while only several chromosomes in *P. yessoensis* showed the clustering brighter signals regions whose coverage and brightness were smaller and weaker than those in *C. farreri*. Moreover, no obvious clustering signals of CF* C_0_t*-1 DNA regions were found in *A. irradians*. 

## 4. Discussion

In bivalves, most comparative cytogenetic studies using FISH have concentrated on some multicopy genes, such as histone and ribosomal RNA genes (rDNAs). Whereas all these studies were relied on the locus of a single gene, and so far there have been no reports about the comparative analysis of highly and moderately repetitive sequences in the whole genomewide in bivalve species. Among repetitive DNAs, *C_0_t*-1 DNA mainly contains highly and moderately repetitive DNA sequences [29]. And *C. farreri* possesses the mode haploid number for Pectinidae (*n* = 19) and the highest number of chromosomal arms (38), which is considered the closest representative of the ancestral karyotype of Pectinidae [13]. Thus, in this study, we used *C_0_t*-1 DNA of *C. farreri* (CF* C_0_t*-1 DNA) as probes to compare the repetitive sequences distribution among *C. farreri*, *P. yessoensis,* and *A. irradians*. We localized the CF* C_0_t*-1 DNA on chromosomes of three scallops by FISH. The results showed that the distributions of highly and moderately repetitive sequences from *C. farreri* not only existed in the genome of *C. farreri, *but also in those of *P. yessoensis *and *A. irradians. *These indicated that the repetitive DNA sequences showed a certain degree of conservation in the process of species evolution. The similar comparative study has also been performed in genus Oryza by Lan et al. [25] and indicated that highly and moderately repetitive sequences in genus Oryza were quite conserved during evolution.

Although highly and moderately repetitive sequences of *C. farreri* existed in the genomes of *C. farreri*, *P. yessoensis,* and *A. irradians*, signal coverage, strength, and area on chromosomes of these three species were different strikingly. We speculated that highly and moderately repetitive sequences are most likely species-specific. Moreover, CF* C_0_t*-1 DNA showed more intensive and brighter signals on the chromosomes in *P. yessoensis* than in *A. irradians, *which may indicate a relatively higher homology in the repetitive DNA sequences between the genome of *P. yessoensis* and *C. farreri*, than that between *A. irradians* and* C. farreri.* The relationship among these three scallops herein are in accordance with the previous molecular studies that used sequences of the mtDNA [4] or internal transcribed spacer region [31], and the classification system based on microsculpture of shell features and morphological characteristics of juveniles [2, 3]. 

Comparative studies on diverse bivalves have shown that chromosome structures are incredibly dynamic in terms of number and location of rDNAs [32–34]. Moreover, Wang and Guo [13] postulated chromosomal translocation and duplication may play a dominant role in the karyotypic evolution of Pectinidae by detecting the major and minor rDNA patterning in *C. farreri* and *A. irradians*. Huang et al. [7] speculated that the nonreciprocal translocation of chromosome with 18S–28S rDNA loci lead to one 18S–28S rDNA site in *C. farreri *into two in *P. yessoensis*. In addition, the comparative chromosomal localization of histone H3 gene in four scallop species (*C. farreri, C. nobilis, P. yessoensis,* and *A. irradians*) suggested that gene duplication/diminution and chromosome rearrangements may have played important roles during chromosome evolution in Pectinidae [14]. In this study, contrasting the similar-dissimilar distribution of CF* C_0_t*-1 DNA on *P. yessoensis* and *C. farreri* chromosomes, we speculate that in the chromosome evolution, highly and moderately repetitive sequences variation, losses, or rearrangement took place in some chromosomes but not all chromosomes. In other words, the evolution of these repetitive sequences was not synchronized between different chromosomes. And the variable distribution patterns of the CF* C_0_t*-1 DNA suggested that repetitive sequences variation, losses, as well as chromosome rearrangements may have played important roles in the genomic evolution of Pectinidae. 

In the present study, our results revealed the distribution of these repetitive DNA sequences in the genome of *C. farreri*. FISH demonstrated that CF* C_0_t*-1 DNA not only dispersed on all chromosomes, but also more densely organized in centromeric, pericentromeric, and telomeric regions of the most chromosomes, which showed clearly fluorescent signal banding. Repetitive sequence regions usually correspond to constitutive heterochromatin in the genome. As has been shown by Chang et al. [35], the FISH of *C_0_t *fractions and of various tandem and dispersed repeats in tomato demonstrated that most of the repeats are confined to the clearly distinguishable heterochromatin blocks at the telomeres, in the pericentromeres and in the large nucleolar organizer region (NOR). In the case of *C. farreri*, 18S–28S rDNA, as well as nucleolar organizer region (NOR) has been located on the short arm of subtelecentric chromosome 10 [6]. A tandem repeats satellite DNA *Cf303* has also been hybridized to the centromeric and telomeric region of the long arm of a pair of subtelocentric chromosomes and the telomeric region of the long arm of 13 pairs of submetacentric or subtelocentric chromosomes [17]. Based on our FISH result of *C_0_t* fractions and the former published FISH data of tandem repeats satellite DNA and rDNA, we can basically distinguish the constitutive heterochromatin regions in the genome of *C. farreri*.

Chromosome identification is the first step in understanding the genome organization of one species. The chromosomes of *C. farreri* show a similarity and continuity except for three pairs of metacentric chromosomes. It is difficult to identify homologues and distinguish different chromosomes to generate an accurate karyotype by traditional karyotype analysis. The application of FISH can help in achieving this target, but FISH with satellite DNA or vertebrate telomere sequence has been demonstrated to be unsuitable for the similar and even identical location of signals on chromosomes in bivalve species [36–38]. Repetitive genes such as rRNA and histone H3 genes can be used for chromosome identification [13, 14], yet these specific probes containing repetitive sequences were very limited. Unique sequences like large-insert clones are easy to achieve and fosmid clones of *C. farreri* have been applied to develop chromosome-specific probes, and 8 of the 19 fosmid clones selected were successfully used for chromosome identification [17]. However, there has not been a successful example to realize the identification of all the 19 chromosome pairs of *C. farreri. *In the present study, the mitotic metaphase chromosome pairs of *C. farreri* could be stably banded by CF* C_0_t*-1 DNA, and the specific and analogous banding patterns were exhibited on the two members of the homologous chromosome pairs (Figure 3). This indicated that the homologous chromosomes possessed homologous or similar highly and moderately repetitive DNA sequences, while nonhomologous chromosome pairs did not. These were the foundation of karyotyping with *C_0_t*-1 DNA banding. This karyotyping method based on *C_0_t*-1 DNA fluorescent bands has been successfully practiced in *Brassica napus* L. [39] and *Brassica oleracea* L. [24]. Compared with conventional karyotype analysis, the karyotyping technique reported in this paper was based on the genome constitution, and therefore it was faster and more exact to match the homologous chromosomes and discriminates different chromosomes. Although the majority of chromosomes were identified based on FISH of CF* C_0_t*-1 DNA, and karyotype analysis was carried out in *C. farreri.* It would be difficult to judge the exact location of CF* C_0_t*-1 DNA on the long arm, the short arm, or the centromeric sites of the chromosomes because the emanative fluorescence signals existing on the pericentromeric sites covered the centromeres. Different chromosomes are still difficult to distinguish when they show very similar banding patterns. Therefore, the technology combining the banding patterns of CF* C_0_t*-1 DNA with the FISH location analysis of specific sequence probes (such as BAC-FISH) would increase the veracity and reliability of chromosome identification in *C. farreri.*
